# Medial Temporal Atrophy Contributes to Cognitive Impairment in Cerebral Small Vessel Disease

**DOI:** 10.3389/fneur.2022.858171

**Published:** 2022-05-18

**Authors:** Wenshan Sun, Lili Huang, Yue Cheng, Ruomeng Qin, Hengheng Xu, Pengfei Shao, Junyi Ma, Zhelv Yao, Lin Shi, Yun Xu

**Affiliations:** ^1^Department of Neurology, Nanjing Drum Tower Hospital, Clinical College of Nanjing Medical University, Nanjing, China; ^2^Department of Neurology, Nanjing Drum Tower Hospital, Medical School and The State Key Laboratory of Pharmaceutical Biotechnology, Institute of Brain Science, Jiangsu Key Laboratory for Molecular Medicine, Nanjing University, Nanjing, China; ^3^Department of Neurology, Affiliated Jiangning Hospital of Nanjing Medical University, Nanjing, China; ^4^Department of Imaging and Interventional Radiology, The Chinese University of Hong Kong, Hong Kong, Hong Kong SAR, China; ^5^BrainNow Research Institute, Hong Kong Science and Technology Park, Hong Kong, Hong Kong SAR, China

**Keywords:** brain atrophy, medial temporal atrophy, cerebral small vessel disease, white matter hyperintensities, cognitive impairment

## Abstract

**Background:**

The role of brain atrophy in cognitive decline related to cerebral small vessel disease (CSVD) remains unclear. This study used AccuBrain™ to identify major CSVD-related brain changes and verified the relationship between brain atrophy and different cognition domains in CSVD patients.

**Methods:**

All enrolled 242 CSVD patients and 76 healthy participants underwent magnetic resonance imaging examinations and detailed neuropsychological scale assessments were collected at the same time. The AccuBrain™ technology was applied to fully automated image segmentation, measurement, and calculation of the acquired imaging results to obtain the volumes of different brain partitions and the volume of WMH for quantitative analysis. Correlation analyses were used to estimate the relationship between MRI features and different cognitive domains. Multifactor linear regression models were performed to analyze independent predictors of MTA and cognitive decline.

**Results:**

CSVD patients exhibited multiple gray matter nucleus volume decreases in the basal ganglia regions and brain lobes, including the temporal lobe (*P* = 0.019), especially in the medial temporal lobe (*p* < 0.001), parietal lobe (*p* = 0.013), and cingulate lobe (*p* = 0.036) compare to HC. The volume of PWMH was an independent predictor of MTA for CSVD patients. Both medial temporal atrophy (MTA) and PWMH were associated with cognition impairment in CSVD-CI patients. MTA mediated the effect of PWMH on executive function in CSVD-CI patients.

**Conclusions:**

Our results showed that MTA was related to cognition impairment in CSVD patients, which might become a potential imaging marker for CSVD-CI.

## Introduction

Cerebral small vessel disease (CSVD) is one of the main causes of vascular cognitive impairment and vascular dementia (VD). The neuroimaging features of CSVD include small subcortical infarcts, lacunes, white matter hyperintensities (WMHs), enlarged perivascular spaces, cerebral microbleeds (CMBs), and brain atrophy (1).

Brain atrophy, especially hippocampal atrophy and medial temporal lobe atrophy (MTA), has been proven to be closely related to Alzheimer's disease (AD) while increasing evidence has shown that it has also been associated with cognitive performance in cerebrovascular diseases alone or in combination with other factors (2–4). The mechanisms of the relationship between brain atrophy and vascular dementia are still unknown, evidence showed that individuals with cognitive dysfunction develop microstructure damage and BBB breakdown in the hippocampus irrespective of Alzheimer's biomarker changes, suggesting that neurovascular dysfunction may represent a factor contributing to cognitive decline, independent of the classic pathophysiological hallmarks of AD (5). Previous studies have reported that the strongest predictor of cognitive performance in patients with CSVD was the volume of WMH (6, 7) which may have a direct effect on cognition by disrupting brain networks sub-serving cognitive processes. Vascular risk factors are strongly associated with WMH, suggesting that the etiology of WMH is more likely related to vascular diseases (8). However, WMH contributes to cognitive decline and neuronal loss not only in VD but also in AD (9, 10). Although the relationship between WMH with cognitive functioning and AD has been described consistently, the mechanisms of this relationship are poorly understood. Therefore, it is important to examine CSVD markers in addition to AD markers in older adults presenting with CSVD. Additionally, the progression of WMH, especially periventricular WMH (PWMH), has a crucial impact on brain atrophy (11). The intermediary role of brain atrophy in cognition decline has been proposed but needs to be further confirmed.

Computer-generated magnetic resonance imaging (MRI) segmentation has been available for different types of CSVD changes. AccuBrain™ is a multi-atlas-based anatomical segmentation tool that has good accuracy in the segmentation of subcortical structures and has been used for the quantification of brain volumetry and volumetric structural covariance. It has been validated for AD to have a better performance among the existing automatic brain segmentation tools (12). This study aimed to use AccuBrain™ to identify major CSVD-related brain imaging features and verify the relationship between brain atrophy and different cognition domain in CSVD patients.

## Methods

### Participants

Two hundred and forty two CSVD patients and 76 healthy individuals among the outpatients and inpatients in the Department of Neurology between January 2017 and January 2019 were consecutively recruited. Ethical approval was provided by the ethics committee of Nanjing Drum Tower Hospital and written informed consent was received from all participants. Based on the established research criteria, CSVD in this study was defined as lesions of moderate-to-severe WMH (Fazekas score of 2 or higher) and/or lacunar infarction (LI) on neuroimaging, with or without perivascular spaces, microbleeds, and brain atrophy (1, 13, 14). WMHs are hyperintense on T2-weighted or FLAIR sequences, appearing as isointense or hypointense on T1-weighted sequences, depending on the sequence parameters and severity of the pathological changes, LI is a small subcortical infarct with a diameter ranging from 3 to 15 mm on axial sections (1).

The inclusion criteria were as follows: (a) age of 45–84 years; (b) CSVD diagnosis; and (c) agreement to sign an informed consent form. The exclusion criteria were as follows: (a) cerebral infarctions >20 mm in diameter; (b) leukoencephalopathy of non-vascular origin (e.g., multiple sclerosis, immunological demyelination, and metabolic, toxic, or infectious diseases); (c) intracranial or extracranial large artery stenosis of >50%; (d) intracranial hemorrhage; (e) other diseases interfering with neuropsychological tests, such as AD, Parkinson's disease, or severe psychiatric disorders; (f) inability or refusal to undergo cerebral MRI, and (g) left-handedness. All control participants underwent a brain MRI scan and had no territorial infarctions or other structural brain lesions on brain MRI.

### MRI Protocol and Image Processing

All participants were studied using MRI following a standard protocol. Scans were obtained using a 3T Philips Intera scanner (Achieva 3.0 T TX, Philips Medical Systems, the Netherlands) at the Imaging Department of Drum Tower Hospital. The protocol included the following sequences: Three-dimensional, high-resolution T1 weighted turbo gradient echo sequence was performed with the following parameters: repetition time (TR) = 9.8 ms, echo time (TE) = 4.6 ms, flip angle (FA) = 8°, slices = 192, the field of view (FOV) = 250 × 250 mm2, acquisition matrix = 256 × 256, thickness = 1.0 mm. The fluid-attenuated inversion recovery (FLAIR) images were performed with TR/TE/inversion time (TI) at 4,500/333/1,600 ms, slices = 200, voxel size = 0.95 × 0.95 × 0.95 mm3, acquisition matrix = 270 × 260. In addition, axial T2-weighted, diffusion-weighted imaging (DWI) sequence, and susceptibility-weighted imaging (SWI) were collected to detect acute or subacute infarctions, and cerebral microbleeds. At baseline, participants underwent brain MRI and clinical assessments, including a standard neurological examination, functional status evaluation, and a neuropsychological examination.

**Figure 1:**
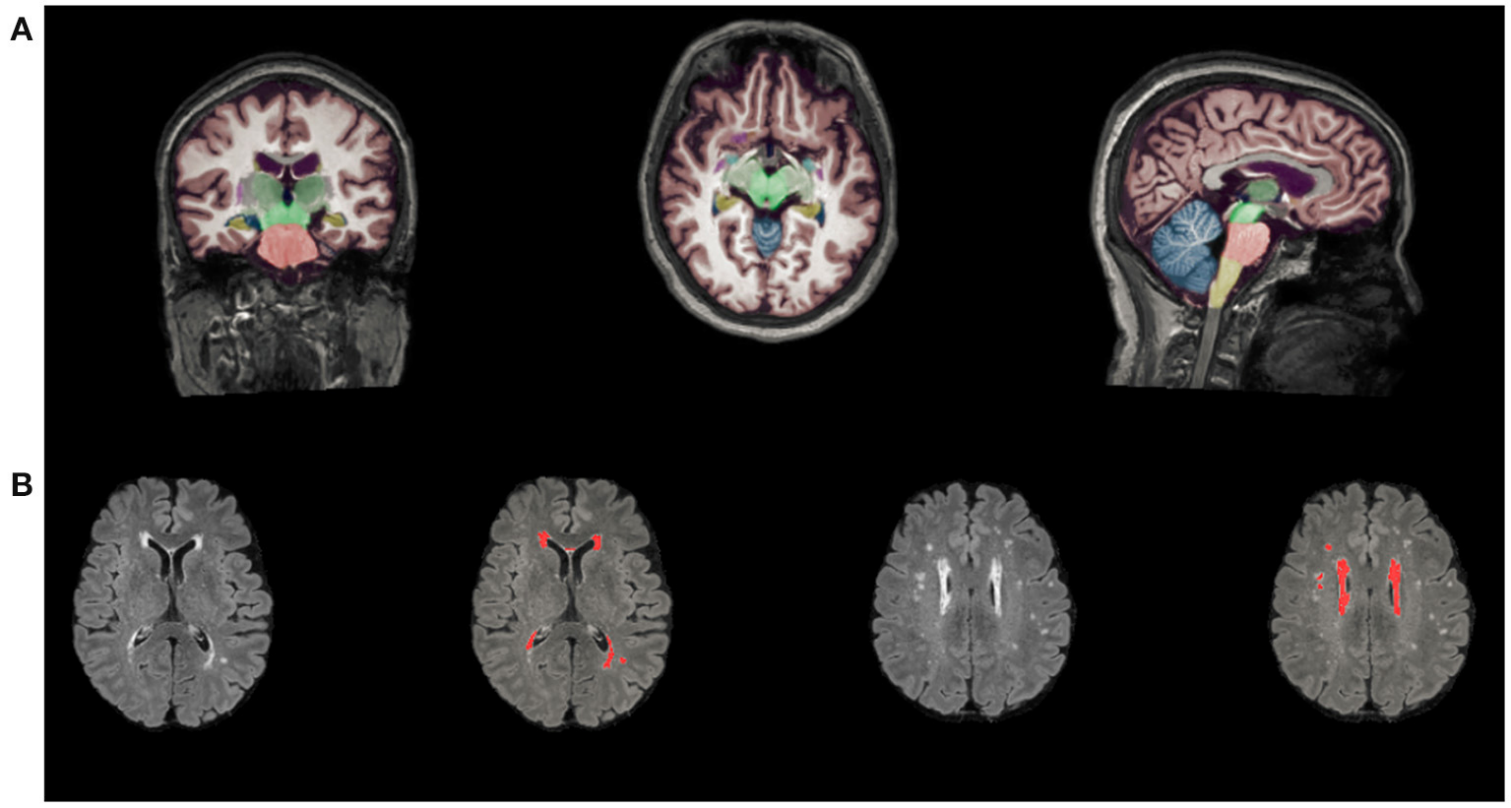
Automated segmentation and quantitative using AccuBrain™. **(A)** Volume segmentation and quantification of anatomical regions based on T1-weighted MRI scans. **(B)** WMH volume segmentation and quantification were based on FLAIR images. WMH, white matter hyperintensities.

The number of LIs (on T1-weighted and FLAIR images) and CMBs (on susceptibility-weighted imaging) was counted by two expert neurologists separately. Volumetry of anatomical regions was obtained from T1-weighted MRI scans automatically segmented using AccuBrain™. The WMH volume segmentation and quantification were based on additional T2-FLAIR MRI images, which were standardized by dividing the volume of different brain regions and WMH volume by the intracranial volume (ICV) for each participant (brain volume/ICV × 100%) Figure 1. MTA is defined as the ratio of the ipsilateral lateral subventricular horn to hippocampal volume.

### Neuropsychological Evaluation

All participants completed neuropsychological measures on the same day as the MRI scan, and overall cognitive function scores were assessed using the Mini-Mental State Examination (MMSE) and Montreal Cognitive Assessment (MoCA). CSVD patients were classified into CSVD-non-CI (CSVD patients without cognitive impairment) and CSVD-CI (CSVD patients with cognitive impairment) groups according to different educational levels, which were presented in our published paper (15). For the evaluation of cognitive subdomains, information processing speed scores were assessed using Stroop Color and Word Tests B (Stroop-B) and Trail Making Test-A (TMT-A). Executive function was obtained via Stroop-C and TMT-B. Contextual memory scores were assessed using the visual reproduction-long-delayed recall portion of the Wechsler Memory Scale (WMS-VR-DR), and the auditory verbal learning test-delayed recall (AVLT-DR) assessment was available. Language scores were obtained through category verbal fluency (CVF) and Boston Naming Test (BNT) assessments. Visuospatial function scores were obtained using the clock drawing test (CDT) and visual reproduction-copy (VR-C) assessments. All raw data were transformed into standard scores (z-score), which were averaged to assess general cognitive function and other cognitive domains.

### Statistical Analyses

Continuous variables with normal distribution were presented as mean ± standard deviation (SD), variables with non-normal distribution are presented as median (interquartile ranges), and categorical as frequencies (percentages). One-way ANOVA was applied for the comparison of normally distributed data, Kruskal–Wallis test was used for the comparisons of non-normal distributed data, and χ^2^ test was applied for the ranked data. A *post hoc* analysis was performed to investigate group differences between any two groups, additionally correcting for multiple comparisons with Bonferroni correction.

Spearman correlation analyses were applied in the CSVD group to assess the relationships between MTA and all other variables of interest, including age, sex, years of education, history of hypertension, history of LI/TIA, LI count, and CMB count, the volume of WMH, PWMH, DWMH. Stepwise multiple linear regression models with MTA as dependent variable and significant factors in the correlation analysis as independent variables controlling for age, sex, years of education, hypertension, and history of TIA were built to determine the relationship between MTA and other conventional MRI markers of CSVD patients. Partial correlation analyses controlling for age, sex, years of education, history of hypertension, and history of LI/TIA were performed in the CSVD-CI group to assess the relationships between cognitive decline and other MRI variables, including LI count, CMB count, WMH volumes, PWMH volumes, DWMH volumes, MTA and regional brain volume. To build predictive models of cognitive functions, a stepwise multiple linear regression analysis was performed in the CSVD-CI patients. The cognitive domain function was taken as the dependent variable, and the significant factors in the correlation analysis were taken as the independent variables.

The PROCESS module (V2.16.3) written by Andrew F. Hayes (www.afhayes.com) was used for the intermediate analysis to explore whether MTA is involved in the relationship between PWMH volume and cognition controlling. First, we tested the direct effects of the primary predictor (PWMH volume) on the mediator (MTA) and the direct relationship between the mediator (MTA) and the outcome (cognitive functioning). Next, we tested the indirect mediating effect on the relationship between PWMH volume and cognitive functioning operating statistically through MTA. We considered PWMH volume, MTA, and cognitive functioning (global and each of the cognitive domains) as predictors, mediators, and outcomes, respectively. We computed bias corrected 95% confidence intervals for the size of the mediating effects with bootstrapping (*k* = 5,000 samples). All data were analyzed using SPSS 23.0 statistical software (Chicago, IL, USA). A *P*-value of <0.05 was considered statistically significant.

## Results

### Demographic, Clinical, and Neuropsychological Characteristics

The demographic and clinical data of the HC and CSVD groups are presented in Table 1. There was no significant difference in gender or prevalence of diabetes mellitus, dyslipidemia, history of coronary heart disease, smoking, and drinking. The CSVD group showed a significantly increased age (*p* = 0.042), history of hypertension (*p* = 0.018), and LI/TIA (*p* =0.001). One hundred and seven CSVD patients were without cognitive impairment (CSVD-non-CI) and 135 with cognitive impairment (CSVD-CI). WMH, PWMH, DWMH volumes, LI count, and CMB count in CSVD-non-CI and CSVD-CI were significantly higher than those in HCs. The CSVD-CI subgroup showed poorer performances on general cognitive function, episodic memory, language, information processing, executive function, and visuospatial function than other subgroups. Compared to CSVD-non-CI group, patients in CSVD-CI group were less educated (*p* = 0.034). No significant differences in the cognitive tests were shown between the CSVD-non-CI group and the HC group.

**Table 1:** Demographic, clinical, volume, and neuropsychological data.

**Item**	**HC (*n* = 76)**	**CSVD**			***F/χ* ^2^ */H***	***p***	***Post hoc*** **analyses**		
		**CSVD-nonCI (*n* = 107)**	**CSVD-CI (*n* = 135)**	**Total (*n* = 318)**			**HC vs. CSVD-non-CI**	**HC vs. CSVD-CI**	**CSVD-non-CI vs. CSVD-CI**
**Demographics**									
Age, years	63 (58, 67.5)	65 (59, 72)	65 (60, 73)	65 (59, 71)	6.36	0.042^*^	0.230	0.037^*^	1.000
Male, *n* (%)	39 (51.3)	59 (55.1)	75 (55.6)	173 (54.4)	0.39	0.824	-	-	-
Education, years	12 (9, 15)	12 (9, 16)	9 (9, 12)	12 (9, 15)	7.07	0.029^*^	1.000	0.240	0.034^*^
**Clinical characteristics**									
Hypertension, *n* (%)	39 (52.3)	75 (70.1)	92 (68.1)	206 (64.8)	8.03	0.018^*^	0.013^*^	0.018^*^	0.781
Diabetes mellitus, *n* (%)	15 (19.7)	28 (26.2)	33 (24.4)	76 (23.9)	1.05	0.592	-	-	-
Hyperlipidemia, *n* (%)	13 (17.1)	23 (21.5)	28 (20.7)	64 (20.1)	0.59	0.745	-	-	-
Coronary heart disease, *n* (%)	6 (7.9)	7 (6.5)	6 (4.4)	19 (6.0)	1.12	0.570	-	-	-
History of LI/TIA, *n* (%)	10 (13.2)	27 (25.2)	49 (36.3)	87 (2.4)	13.21	0.001^*^	0.042^*^	<0.001^*^	0.098^*^
History of smoking, *n* (%)	12 (15.8%)	24 (22.4%)	36 (26.7%)	72 (22.6)	3.29	0.193	-	-	-
History of drinking, *n* (%)	9 (11.8%)	22 (20.6%)	26 (19.3%)	57 (17.9)	2.58	0.275	-	-	-
LI count, *n*	0 (0, 0)	1 (0, 2)	1 (0, 3)	0 (0, 2)	56.50	<0.001^*^	<0.001^*^	<0.001^*^	1.000
CMB count, *n*	0 (0, 0)	0 (0–2)	0.5 (0, 2)	0 (0, 2)	36.51	<0.001^*^	<0.001^*^	<0.001^*^	0.284
**Volume data**									
ICV (mL)	1425.85 ± 116.40	1435.29 ± 124.84	1425.55 ± 129.62	1428.90 ± 124.66	0.21	0.810	-	-	-
Brain parenchyma (mL)	1065.90 ± 88.57	1064.17 ± 95.20	1045.69 ± 102.11	1056.74 ± 96.88	1.54	0.217	-	-	-
Hippocampus (mL)	6.87 ± 0.65	6.94 ± 0.72	6.72 ± 0.79	6.84 ± 0.74	2.77	0.064	-	-	-
Amygdala (mL)	3.62 ± 0.39	3.70 ± 0.45	3.63 (3.35, 3.87)	3.64 (3.35, 3.89)	1.69	0.430	-	-	-
Thalamus-Proper (mL)	12.12 ± 1.01	11.98 ± 1.09	11.72 ± 1.14	11.91 ± 1.10	3.87	0.022^*^	0.998	0.025^*^	0.210
Caudate (mL)	6.55 ± 0.72	6.93 ± 0.82	6.96 (6.41, 7.60)	6.81 (6.29, 7.43)	16.27	<0.001^*^	0.004^*^	<0.001^*^	1.000
Putamen (mL)	10.61 ± 1.01	10.84 ± 0.99	10.80 ± 1.29	10.77 ± 1.13	1.01	0.364	-	-	-
Pallidum (mL)	3.06 ± 0.35	3.04 (2.74, 3.23)	2.97 ± 0.34	2.99 ± 0.35	2.24	0.327	-	-	-
Hypothalamus (mL)	0.64 (0.60, 0.71)	0.66 ± 0.07	0.65 ± 0.07	0.65 ± 0.07	0.84	0.660	-	-	-
MTA	0.35 ± 0.06	0.38 (0.33, 0.48)	0.43 (0.35, 0.54)	0.3 (0.33, 0.48)	38.89	<0.001^*^	0.001^*^	<0.001^*^	0.014^*^
White matter (mL)	486.07 ± 47.70	485.41 ± 52.58	480.30 ± 53.38	483.40 ± 5,172	0.42	0.655	-	-	-
WMH (mL)	1.57 ± 0.64	3.87 (1.74, 7.62)	4.9 (2.65, 11.86)	3.15 (1.60, 6.62)	88.45	<0.001^*^	<0.001^*^	<0.001^*^	0.046^*^
PWMH (mL)	1.18 ± 0.61	2.97 (1.41, 6.3)	4.33 (2.10, 10.82)	2.51 (1.18, 5.96)	91.28	<0.001^*^	<0.001^*^	<0.001^*^	0.025^*^
DWMH (mL)	0.35 (0.18, 0.55)	0.49 (0.25, 0.96)	0.53 (2.63, 1.05)	0.44 (0.25, 0.86)	17.13	<0.001^*^	0.004^*^	<0.001^*^	1.000
Gray matter (mL)	579.82 ± 46.57	578.75 ± 48.61	565.39 ± 54.02	573.34 ±50.83	2.91	0.056	-	-	-
Frontal lobe (mL)	151.10 ± 13.48	150.47 ± 15.08	147.36 ± 15.83	149.30 ± 15.09	1.99	0.138	-	-	-
Occipital lobe (mL)	63.43 ± 6.77	62.38 ± 6.90	61.09 ± 8.16	62.08 ± 7.47	2.54	0.080	-	-	-
Temporal lobe (mL)	97.31 ± 9.05	98.07 ± 10.71	94.53 ± 10.43	96.39 ± 10.3	3.99	0.019^*^	1.000	0.178	0.023^*^
Parietal lobe (mL)	82.13 ± 7.69	81.85 ± 7.28	79.30 ± 8.64	80.83 ± 8.06	4.37	0.013^*^	1.000	0.042^*^	0.042^*^
Cingulate lobe	23.77 ± 2.39	23.80 ± 2.56	23.03 ± 2.64	23.47 ± 2.57	3.37	0.036^*^	0.939	0.046^*^	0.022^*^
Insular (mL)	12.39 ± 1.28	12.34 ± 1.44	12.12 ± 1.48	12.26 ± 1.42	1.10	0.335	-	-	-
**Neuropsychological data**									
General cognitive function	0.59(0.27, 0.85)	0.59 (0.27,0.76)	−0.35 (−1.07,0.00)	0.14(-0.31,0.63)	150.03	<0.001^*^	1.000	<0.001^*^	<0.001^*^
Episodic memory	0.38 ± 0.74	0.27 ± 0.88	−0.34 ± 0.79	0.03 ± 0.80	29.91	<0.001^*^	1.000	<0.001^*^	<0.001^*^
Language	0.24 ± 0.72	0.27 ± 0.70	−0.37 ± 0.74	0.00 ± 0.86	19.01	<0.001^*^	1.000	<0.001^*^	<0.001^*^
Information processing speed (inverse)	−0.33 (−0.49, −0.11)	−0.35 (−0.49, −0.10)	0.06 (−0.24, 0.50)	−0.21 (−0.42,1.58)	67.97	<0.001^*^	1.000	<0.001^*^	<0.001^*^
Executive function (inverse)	−0.33 (−0.49, −0.11)	−0.34 (−0.52, −0.08)	0.02 (−0.27, 0.55)	−0.20 (−0.43,1.12)	58.27	<0.001^*^	1.000	<0.001^*^	<0.001^*^
Visuospatial function	0.41 (0.41, 0.41)	0.41 (0.41,0.41)	0.41 (−0.41, 0.41)	0.41 (0.00,0.41)	18.47	<0.001^*^	1.000	0.015^*^	<0.001^*^

*Values are presented as the mean ± standard deviation (SD), median (interquartile ranges) or number (percentage). One-way ANOVA was applied for the comparison of normally distributed data, χ2 test was applied for the ranked data, and the Kruskal–Wallis test was used for the comparisons of non-normally distributed data*.

* *Indicates a statistical difference between groups, p < 0.05*.

*HC, health control; CSVD, cerebral small vessel disease; CSVD-nonCI, CSVD patients without cognitive impairment; CSVD-CI, CSVD patients with cognitive impairment; n, number; TIA, transient ischemic attack; ICV, intracranial volume; MTA, medial temporal atrophy, the ratio of the ipsilateral lateral subventricular horn to hippocampal volume. WMH, white matter hyperintensities; WMH, total white matter hyperintensities; PWMH, periventricular white matter hyperintensities; DWMH, deep white matter hyperintensities; LI, lacunar infarction; CMB cerebral microbleed*.

### Group Comparisons of Brain Regional Volumetry Quantified Using AccuBrain™

The group comparisons of brain volumetric differences quantified using AccuBrain^TM^ are also summarized in Table 1. The volume of the thalamus-proper, caudate, temporal lobe, parietal lobe, cingulate lobe, and MTA were statistically significant among groups. As the *post-hoc* analysis showed, compared with the CSVD-non-CI group, the CSVD-CI had severer atrophy in the temporal lobe (*p* = 0.023), parietal lobe (*P* = 0.042), and cingulate lobe (*p* = 0.022). Notably, MTA exhibited significant differences between HC vs. CSVD-non-CI (*p* = 0.001), HC vs. CSVD-CI (*p* < 0.001), and CSVD-non-CI vs. CSVD-CI (*p* = 0.014).

### Association Between MTA and Conventional MRI Markers of CSVD Patients

In CSVD patients, Spearman correlation analyses showed MTA were significantly correlated to age (*r* = 0.524, *p* < 0.001), WMH volume (*r* = 0.436, *p* = p < 0.001), PWMH volume (*r* = 0.472, *p* < 0.001), LI count (*r* = 0.138, *p* = 0.035), and CMB count (*r* = 0.230, *p* < 0.001). No significant correlation was found between MTA and DWMH volume (*r* = 0.046, *p* = 0.475). Age, sex, education and hypertension and history of TIA, LI count, and CMB count, PWMH volume was further included in the stepwise multiple linear regression models with MTA as dependent variable. Multiple linear regression analysis revealed that the volume of PWMH was an independent predictor of MTA for CSVD patients (β = 0.478, 95%CI: 0.377–0.581).

### Associations Between Brain Atrophy and Cognition in CSVD-CI Patients

For 135 CSVD-CI patients, partial correlation analyses revealed that volume of WMH and PWMH, MTA, CMB count, volume of the temporal lobe, parietal lobe, and cingulate lobe were significantly associated with impairment in various cognitive domains controlling for age, sex, years of education, history of hypertension, and history of LI/TIA.

**Figure 2:**
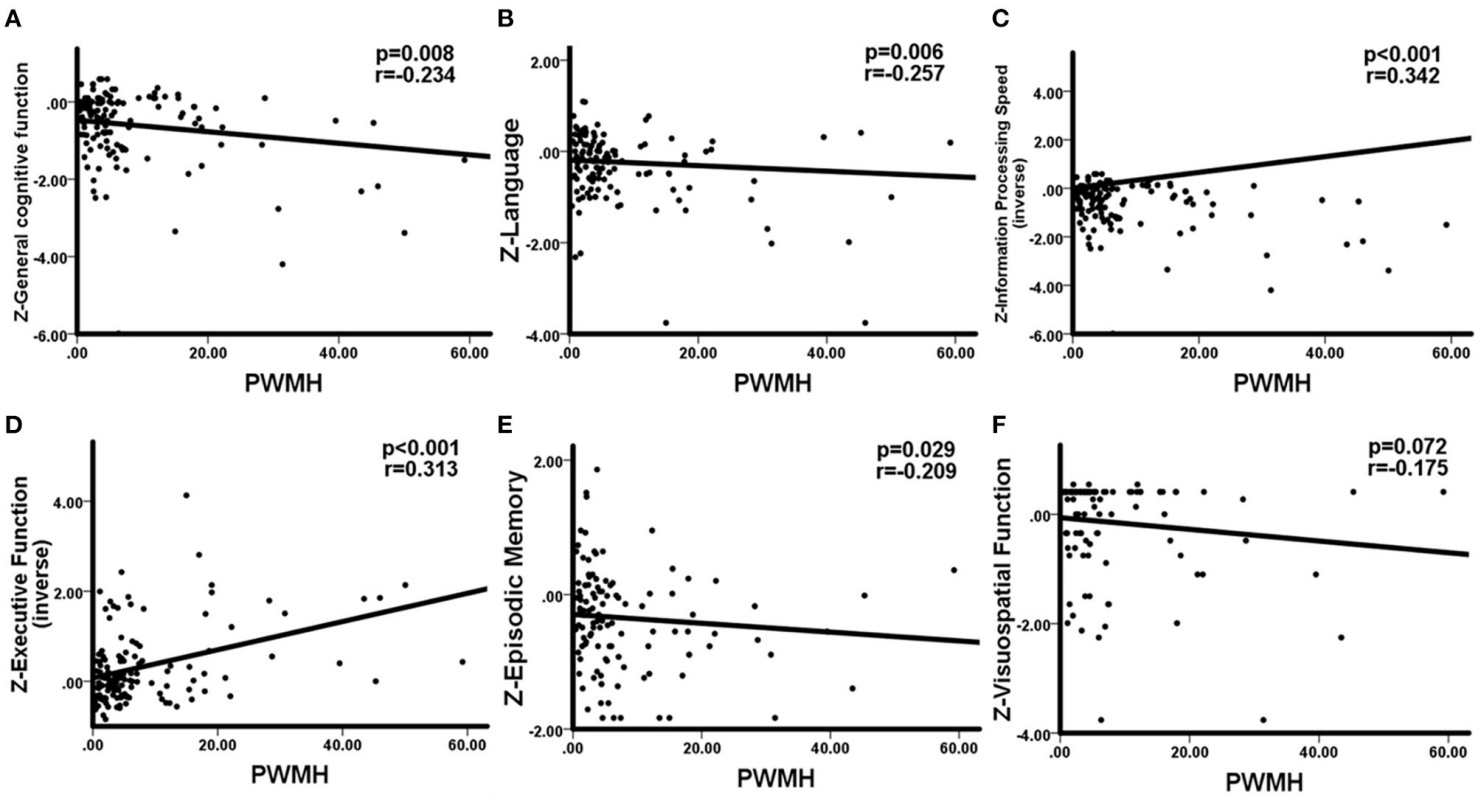
Correlations between PWMH and cognitive function in CSVD-CI patients. Partial correlation was conducted by controlling for age, gender, years of education, history of hypertension, and history of LI/TIA in CSVD-CI group. **(A)** Increased PWMH volume was associated with worse general cognitive function. **(B)** Larger PWMH volume showed significant impairment in the language domain. **(C)** Information processing speed was negatively associated with increased PWMH volume. **(D)** PWMH volume had a negative correlation with executive function. **(E)** PWMH volume correlated negatively with episodic memory. **(F)** No significant correlation was observed between PWMH volume and visuospatial function. PWMH, periventricular white matter hyperintensities; CSVD-CI, cerebral small vessel disease patients with cognitive impairment; TIA, transient ischemic attack; LI, lacunar infarction.

**Figure 3:**
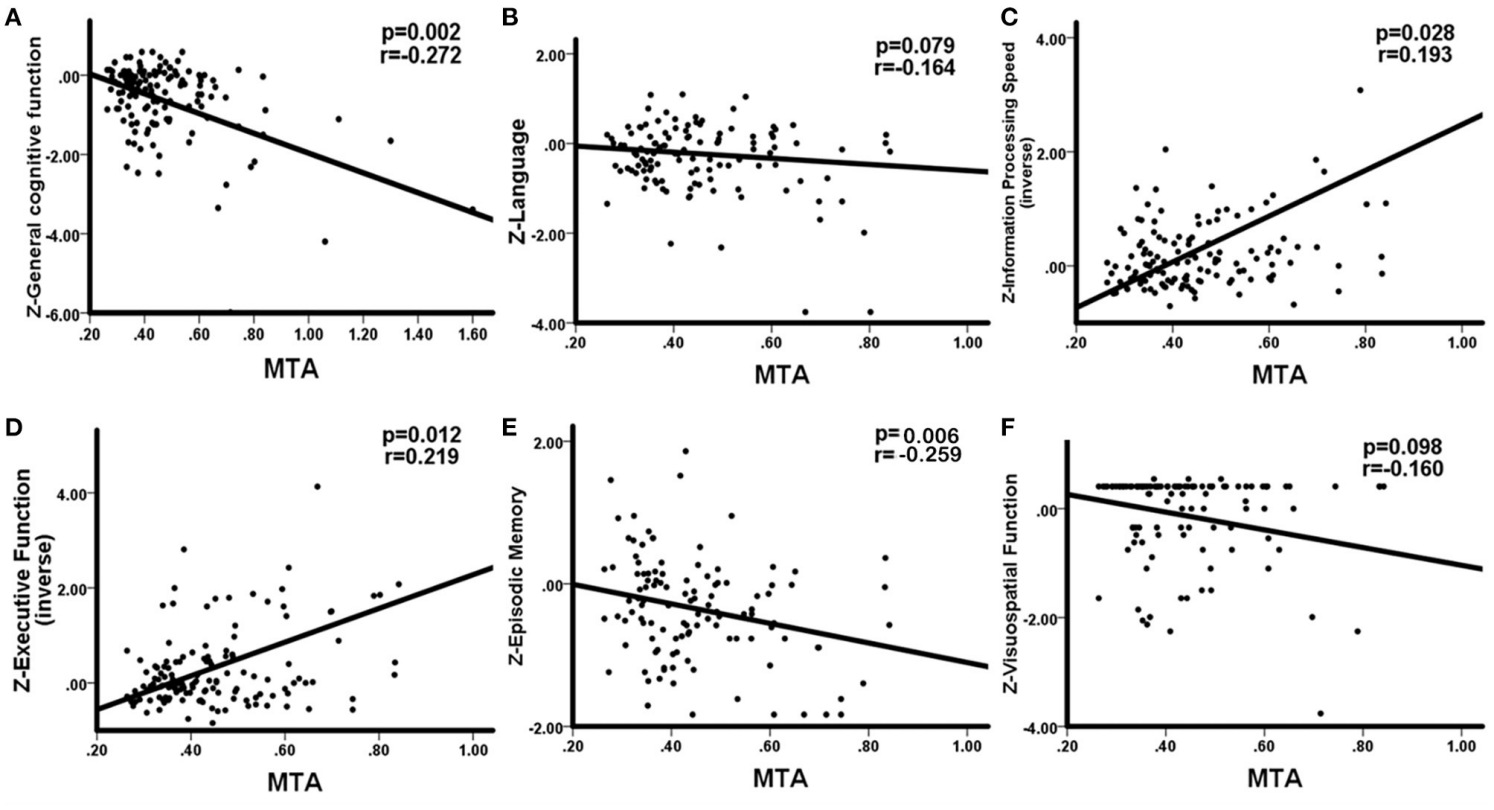
Correlations between MTA and cognitive function in CSVD-CI patients. Partial correlation was conducted by controlling for age, gender, years of education, history of hypertension, and history of LI/TIA in CSVD-CI group. **(A)** MTA correlated negatively with general cognitive function. **(B)** No significant correlation was observed between MTA and the language domain. **(C)** MTA had a negative correlation with information processing speed. **(D)** MTA had a negative correlation with executive function. **(E)** MTA correlated negatively with episodic memory. **(F)** No significant correlation was observed between MTA and visuospatial function. MTA, medial temporal atrophy, the ratio of the ipsilateral lateral subventricular horn to hippocampal volume; CSVD-CI, cerebral small vessel disease patients with cognitive impairment; TIA, transient ischemic attack; LI, lacunar infarction.

WHM volume was negatively correlation with general cognitive function (*r* = −0.224, *p* = 0.011), language (*r* = −0.266, *p* = 0.004), information processing speed (*r* = −0.353, *p* < 0.001), executive function (*r* = −0.310, *p* < 0.001), and memory (*r* = −0.191, *p* = 0.046). No significant correlations between DWMH and cognition were observed. The correlations between PWMH volume and cognitive function are presented in Figure 2, correlations between MTA and cognitive function are presented in Figure 3. In summary, PWMH and MTA extensively affected various cognitive domains in CSVD-CI patients. Particularly, PWMH volume was associated with impairment in all cognitive domains except visual space. MTA had a more prominent effect on executive function and information processing speed, and memory than on language and visuospatial function.

We found the CMB count was significantly related to language (*r* = −0.188, *p* = 0.046), information processing speed (*r* = −0.224, *p* = 0.011), executive function (*r* = −0.201, *p* = 0.023) but not general cognitive function (*r* = 0.071, *p* = 0.429), memory (*r* = −0.047, *p* = 0.629) and visuospatial function (*r* = −0.070, *p* = 0.478). No significant correlations existed between LI count and cognition. Both temporal lobe volume and parietal lobe volume was positively correlated with information processing speed (*r* = 0.233, *p* = 0.008; *r* = 0.192, *p* = 0.028) and executive function (*r* = 0.258, *p* = 0.003; *r* = 0.252, *p* = 0.004). Positive association was also observed between cingulate lobe volume and information processing speed (*r* = 0.175, *p* = 0.046).

In multiple linear regression analysis, general and each cognitive domain was set as dependent variables separately, the volume of PWMH, temporal lobe, parietal lobe, and cingulate lobe, CMB count, and MTA as independent variables, with age, sex, years of education, history of hypertension, history of stroke/TIA as covariant. MTA was significantly associated with worse overall performance in all cognitive domains (Table 2). The volume of PWMH was an independent predictor of decline in executive function. Years of education predicted overall cognitive function, language, memory, executive function, and visuospatial function but not information processing speed. Gender differences were the predictors of overall cognitive and language function. Age was a risk factor for visuospatial deficits. Additionally, cingulate atrophy was predictive of overall cognitive decline; however, no correlation was found with other cognitive domains.

**Table 2:** Multiple linear regression analysis for cognitive function in CSVD-CI group (*n* = 135).

	**β**	**95%CI**	***p***
**General cognitive function**			
MTA	−0.558	−0.672 to −0.401	<0.001
Education	0.428	0.290 to 0.555	<0.001
Gender	−0.252	−0.387 to −0.104	0.001
Cingulate lobe	−0.163	−0.309 to −0.014	0.032
**Episodic memory**			
MTA	−0.335	−0.512 to −0.159	<0.001
Education	0.184	0.006 to 0.297	0.041
**Language**			
MTA	−0.325	−0.417 to −0.135	<0.001
Education	0.282	0.100 to 0.390	0.001
Gender	−0.196	−0.288 to −0.023	0.020
**Information processing speed (inverse)**			
MTA	0.448	0.339 to 0.702	<0.001
**Executive function (inverse)**			
MTA	0.350	0.180 to 0.582	<0.001
Education	−0.221	−0.413 to −0.079	0.004
PWMH volume	0.207	0.025 to 0.426	0.025
**Visuospatial function**			
Education	0.210	0.032 to 0.383	0.021
MTA	−0.347	−0.660 to −0.196	<0.001
Age	0.263	0.071 to 0.449	0.007

*CSVD, cerebral small vessel disease; CSVD-CI, CSVD patients with cognitive impairment; MTA, medial temporal atrophy, the ratio of the ipsilateral lateral subventricular horn to hippocampal volume; PWMH, periventricular white matter hyperintensities; **β**, standardized coefficients*.

**Figure 4:**
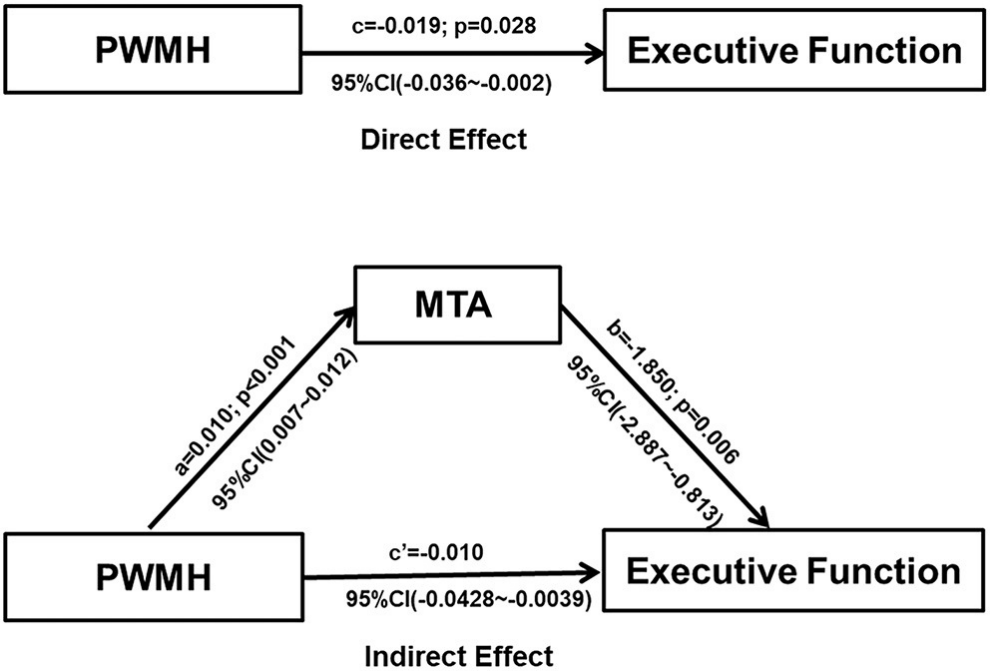
Graphical illustration of the direct and indirect effects of PWMH volume and MTA on cognitive functioning. Each path of the connection, standard coefficient (a, b, c, c'), and *p*-value were shown. Mediation analysis revealed that the association between PWMH and executive function was mediated by MTA (Indirect effect: −0.01, 95% CI: −0.0428 to −0.0039). PWMH, periventricular white matter hyperintensities; MTA, medial temporal atrophy, the ratio of the ipsilateral lateral subventricular horn to hippocampal volume.

Direct and mediated effects of periventricular WMH on executive function are presented in Figure 4. The PWMH had a direct effect on the impairment of executive function (direct effect: −0.019, 95%CI: −0.036~-0.002); a larger PWMH volume was associated with lower executive function performance and more serious MTA. In mediation analyses, the associations of PWMH with executive functioning were significantly mediated by MTA (indirect effect: −0.010, 95%: −0.0428 to−0.0039).

## Discussion

In our study, segmentation and detection of the volumes of brain regions revealed that CVSD showed cerebral atrophy not only in the basal ganglia region but also in the lobes. We further analyzed the factors associated with cognitive decline, and our results revealed that the volume of PWMH and MTA were independent predictors of cognitive decline in CSVD. Previous studies have suggested that WMH is the most common cause of vascular cognitive impairment, from mild cognitive impairment to VD (16), and significantly affects executive function and processing speed (17). Moreover, different areas of WMH distribution contribute to each cognitive domain; the periventricular WMH is more correlated with patients' executive function decline than DWMH, and WMH in the parietal temporal lobe is more correlated with memory decline (18).

Notably, compared with the HC group, the CSVD group showed more WHM volume, both in PWMH and DWMH volumes, while in CSVD subgroup, no significant difference was detected between CSVD-CI and CSVD-non-CI in DWMH volumes. Furthermore, the following correlation analysis also failed to find the association between different cognitive domains and DWMH volumes. PWMH and DWMH were reported to be associated with different histopathological, and aetiological features. From the aspect of histopathologic correlates, peri-WMH mostly reflects non-ischemic damages whereas DWMH is associated with ischemic tissue damages (19). Previous studies found that the elderly with PWMH, rather than DWMH, is associated with impaired cognitive function, especially executive function. A possible explanation was that PWMH lesions interfere with long connections, leading to worse performance in mostly cognitive domains whereas DWMH damage is responsible for short connections that are less associated with cognitive performance but may play an important role in motor dysfunction (20).

Our most important findings are that MTA is an independent risk factor for cognitive decline in CSVD and that the effect of PWMH on cognitive decline is either directly or indirectly mediated by MTA. MTA and hippocampal atrophy have been implicated in age-related cognitive decline and as important imaging markers of AD (21). While it has been also reported that MTA correlates with VD, Arba et al. assessed the relationship between cognition and imaging features in patients with stroke or TIA over a 1-year period and found that moderate to severe MTA was present in 44% of the patients, confirming that VD features are independently associated with MTA (22). In the case of patients with cerebral autosomal dominant arteriopathy with subcortical infarcts and leukoencephalopathy, hippocampal atrophy is significantly associated with dementia (23), and animal models of long-term hypoperfusion have shown that neurodegenerative changes are not a prerequisite for hippocampal atrophy (24). All these findings suggest that MTA is not only an important alteration in neurodegenerative disease but also an important pathway for the development of vascular cognitive dysfunction.

Although the specific mechanisms of cognitive decline in VD remain unclear, MTA and WMH are common morphological features of AD and CSVD. Our study found strong direct effects and indirect effects of WMH on cognition through MTA in CSVD-CI patients, suggesting that some of the consistently observed associations of WMH with cognition are at least partially attributable to their effect on brain atrophy. This finding has implications for understanding why WMH are strong predictors of cognitive decline and AD. Indeed, previous work has shown that in individuals with higher levels of WMH, the coupling of structural and functional connections is disrupted, with subsequent effects on executive function and memory (25). The observed indirect effects of WMH on cognition through cortical thinning may reflect axonal damage associated with CSVD (26), which promotes neurodegenerative changes in the cerebral cortex that in turn drives cognitive loss. This process may be dependent on hyper-phosphorylated tau, suggesting a direct causal link between CSVD and AD pathology (27).

Our study features a combination of big data applications and neuroimaging studies and the quantitative analysis of brain atrophy by applying AccuBrain™ technology, which is an application of machine/deep learning strategy for cerebrovascular disease risk assessment. Further development of the technology will have a greater potential in research related to imaging for CSVD. However, this study had some limitations. First, this is a cross-sectional study, the conclusions will be more convincing if they can be replicated in future longitudinal studies, especially in the exploration of the regional brain atrophy progression with the development of CSVD. Second, due to the small sample size, statistical power might be insufficient. the results need to be further validated with a larger sample. Third, the study had a single assessment method and did not incorporate other functional brain imaging tools, for example, magnetic resonance-based diffusion tensor imaging (DTI) is not involved in this study, which is a powerful non-invasive imaging technique and a very advanced quantitative measurement that can be used to trace white matter microstructures and abnormal white matter connectivity *in vivo*. Last but not the least, we mainly focused on superficial imaging characteristics, the volume of WHM, and brain region, other factors like white matter microstructural, enlarged perivascular spaces on cognition were not analyzed, the potential pathogenesis needs to be further explored in subsequent studies.

In conclusion, brain atrophy in patients with CSVD was mainly characterized by alterations of multiple gray matter nuclei in the basal ganglia regions, including the thalamus and caudate nucleus. Atrophy of the lobes was concentrated in the temporal lobes, especially in the medial temporal lobe. The PWMH was an independent predictor of MTA that independently influences cognitive decline in patients with CSVD. The effect of PWMH on executive function was mediated by MTA.

## Data Availability Statement

The raw data supporting the conclusions of this article will be made available by the authors, without undue reservation.

## Ethics Statement

The studies involving human participants were reviewed and approved by Ethics Committee of Nanjing Drum Tower Hospital. The patients/participants provided their written informed consent to participate in this study. Written informed consent was obtained from the individual(s) for the publication of any potentially identifiable images or data included in this article.

## Author Contributions

YX conceived and designed the analysis. WS performed the analysis and wrote the paper. LH collected and evaluated the brain MRI and also conducted the statistical analysis. YC and RQ evaluated all neuropsychological scales. PS, JM, and ZY collected the clinical characteristics data. LS contributed the analysis tool of segmentation and detection of the volumes of brain structure by AccuBrainTM. All authors contributed to the article and approved the submitted version.

## Conflict of Interest

LS is the director of BrainNow Research Institute. The remaining authors declare that the research was conducted in the absence of any commercial or financial relationships that could be construed as a potential conflict of interest.

## Publisher's Note

All claims expressed in this article are solely those of the authors and do not necessarily represent those of their affiliated organizations, or those of the publisher, the editors and the reviewers. Any product that may be evaluated in this article, or claim that may be made by its manufacturer, is not guaranteed or endorsed by the publisher.
